# Autopsy findings in a patient with primary systemic AL (kappa light chain) amyloidosis

**DOI:** 10.4322/acr.2021.273

**Published:** 2021-05-06

**Authors:** Felipe Lourenço Ledesma, Jussara Bianchi Castelli

**Affiliations:** 1 Universidade de São Paulo (USP), Faculdade de Medicina, Departamento de Patologia, São Paulo, SP, Brasil; 2 Universidade de São Paulo (USP), Hospital das Clínicas, Divisão de Anatomia Patológica, São Paulo, SP, Brasil; 3 Grupo Fleury Medicina e Saúde, São Paulo, SP, Brasil

**Keywords:** Amyloidosis, Multiple Organ Failure, Pneumatosis Cystoides Intestinalis, Tandem Mass Spectrometry, Diagnosis, Autopsy

## Abstract

First described by Rokitansky in 1842, and further characterized by Virchow in 1854, amyloidosis is a disorder caused by amyloid deposition, a fibrillary insoluble protein. The clinical spectrum of amyloidosis is broad, as the amyloid deposition may virtually occur in all tissues. Herein, we report the case of a 66-year-old man with a long-lasting emaciating disease, diagnosed, at autopsy, with primary systemic amyloidosis. Amyloid protein deposition was found in many tissues and organs. The involvement of the vessels’ wall rendered ischemic injury most prominent in the intestinal loops causing mesenteric ischemia. Despite the thorough organic involvement, the immediate cause of death was aspiration bronchopneumonia. Massive amyloid deposition was found in virtually all major organs, such as the heart, liver, kidneys, spleen, pancreas, adrenals, prostate, skin, and thyroid: the latter, a complication of the amyloidosis known as amyloid goiter. Post-mortem review of the deceased’s laboratory workup showed a slightly abnormal kappa:lambda ratio in the blood; however, no clonal lymphoplasmacytic disorder was confirmed in the bone marrow and other lymphoreticular system organs either by the microscopic examination and immunohistochemical staining. Laser-capture microdissection and tandem mass spectrometry of the splenic tissue detected a peptide profile consistent with an immunoglobulin Kappa light chain. The presence of amyloid purpura favors the diagnosis of primary systemic amyloidosis.

## INTRODUCTION

In 1842, Karl von Rokitansky (1804-1878) described the gross appearance of the “waxy or lardaceous aspect of the viscera”, which was further better characterized as “amorphous and hyaline deposits” by Rudolph Virchow (1821-1902) in 1854 with the aid of the light microscopy. He preferred the word “amyloid” to the commonly used terms “waxy” or “lardaceous” changes because of the peculiar reaction with iodine.

Currently, 36 types of fibrillar proteins that form amyloid deposits are described, which generate small fibers that clump together in an identical and insoluble physical structure. Regardless of the clinical situation or chemical composition, once deposited, there is no metabolic pathway for their removal, and as the organism is unable to eliminate them, they gradually deposit and damage organs and systems.^1^^-^^4^

The amyloidosis can be hereditary or acquired in etiology and systemic or localized in distribution. The localized form is usually associated with the deposition of immunoglobulin light chain (AL type), although serum amyloid A protein and transthyretin have been reported as causing localized disease, and it commonly involves the laryngo-tracheobronchial tree, urogenital tract, and skin.^5^

Systemic amyloidosis results from the accumulation of amyloid in a wide range of tissues and organs. The primary systemic amyloidosis occurs in association with the so-called plasma cell dyscrasias, a monoclonal proliferation of plasma cells that produce a clonal immunoglobulin protein, i.e., monoclonal gammopathies or multiple myeloma, as well as other malignant B-cell lymphoproliferative disorders with paraproteinemia, i.e., Waldenström's macroglobulinemia. The secondary form of systemic amyloidosis occurs in the presence of preceding or coexisting chronic inflammatory or infectious conditions. It is now well understood why different serum amyloidogenic proteins may transform into amyloid fibrils with anti-parallel beta-sheet conformation that accumulate in different sites and cause the disease.^6^^-^^9^

The clinical spectrum of amyloidosis is broad as the amyloid deposits may involve virtually all organs in different degrees. In the heart, the amyloid deposits in the myocardium and causes restrictive cardiomyopathy. In the liver, the massive amyloid deposition in the portal tracts and sinusoids may be associated with hepatic dysfunction and portal hypertension. In the kidneys, the glomerular amyloid deposition initially causes asymptomatic proteinuria, but, as the disease progresses, the renal failure ensues. In the gastrointestinal tract, the clinical manifestations vary and include abdominal pain, malabsorption, and bleeding. Nonspecific symptoms, such as weight loss, are also present in the systemic form.^10^^-^^15^ The differential diagnosis of systemic amyloidosis is wide and depends on the presenting clinical features, which can be hepatosplenomegaly, heart failure, hepatic dysfunction or altered hepatic enzymes, urinalysis alterations with or without renal dysfunction, and skin alterations.^16^

Although it does not define the type of protein deposited, the Congo red stain is still the method for making the diagnosis of amyloidosis on tissue specimens. However, defining the type of amyloidosis is crucial as the current treatment of systemic amyloidosis directly depends on the molecular type of the amyloid protein. The proper diagnosis demands the correlation between signs and symptoms, familial history, genetic workup, histology, immunohistochemistry, Western blot, and proteomics techniques. Currently, mass spectrometry (proteomic) is referred to as a powerful new diagnostic technique for amyloidosis capable of defining which protein is deposited, which can be performed on the same material of the biopsy used for the tissue demonstration of amyloidosis.^17^^,^^18^

## CASE REPORT

A 66-year-old black male was admitted complaining of an unexplained weight loss of 30 kg, front neck lump, purpuric lesions over the trunk and limbs, and proximal weakness over the past year. The laboratory workup was consistent with hepatic dysfunction with mild elevation of the hepatic enzymes, renal failure (creatinine clearance of 40 mL/min) with mild proteinuria, normocytic and normochromic anemia, and thrombocytopenia. Serum protein electrophoresis showed hypoalbuminemia and no abnormality in the gamma-globulin zone. The thyroid function was normal. Serologic testing for HIV and syphilis were negative. The rheumatoid factor and antinuclear antibody were within normal limits. The initial working diagnosis was systemic vasculitis, and the patient was empirically prescribed prednisone without clinical response. After two weeks of hospitalization, the patient started with intermittent abdominal pain and hemodynamic instability. He was transferred to the intensive care unit (ICU) due to severe hypotension. Unfortunately, the outcome was unfavorable, and he died after five days, despite the advanced cardiac and respiratory life support. An autopsy was performed with the consent of the family.

## AUTOPSY FINDINGS

**Figure 1:**
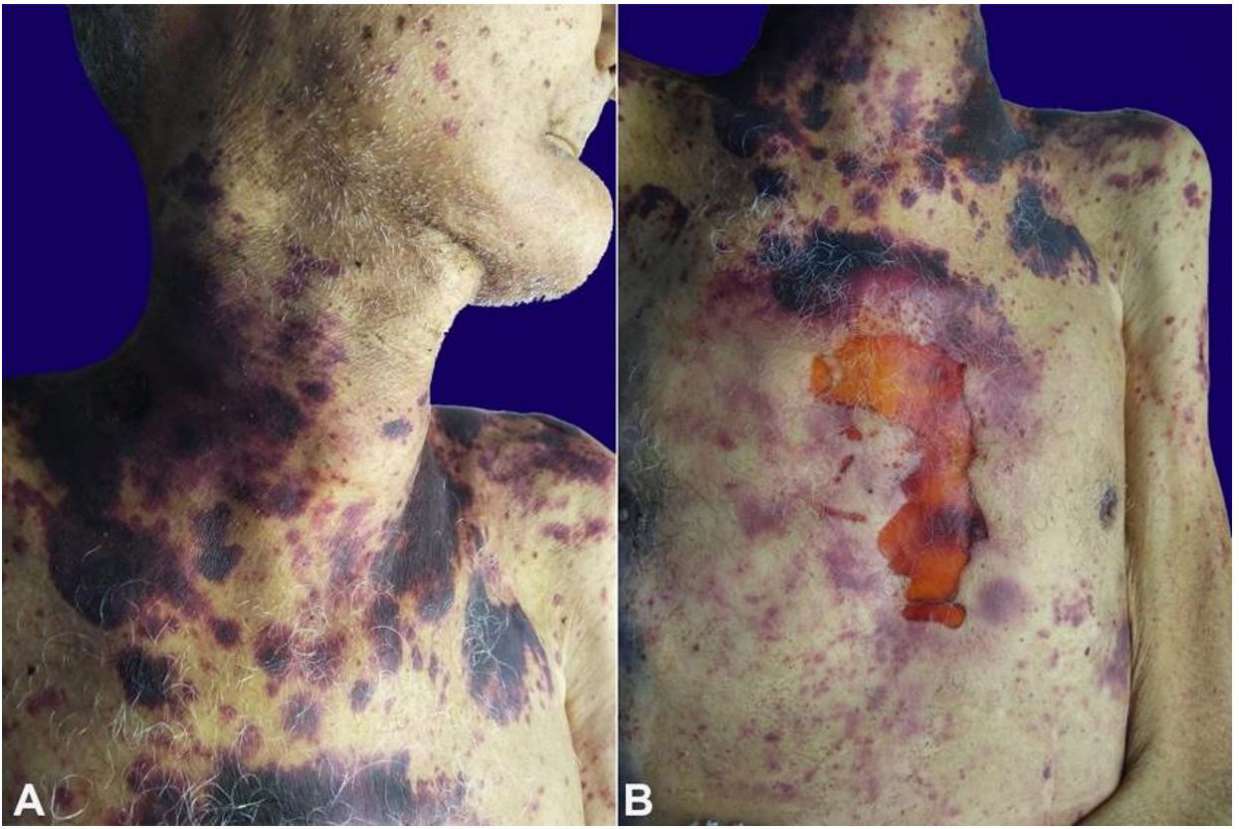
External view of the corpse. **A –** Purpuric cutaneous lesions scattered on the chest and neck – “amyloid purpura”; **B –** Exulcerated lesion in the chest due to the skin friability.

The corpse weighed 65 kg, and the height was 1,75 m (BMI of 21.2 kg/m^2^). On the external examination, the corpse was emaciated. A thyroid goiter and several purpuric lesions in the neck, trunk, and proximal extremity of both arms were easily observed. The skin was friable, so much so that a sizeable hematic crust in the sternal region was attributed to the cardiac resuscitation maneuver (Figure 1).

**Figure 2:**
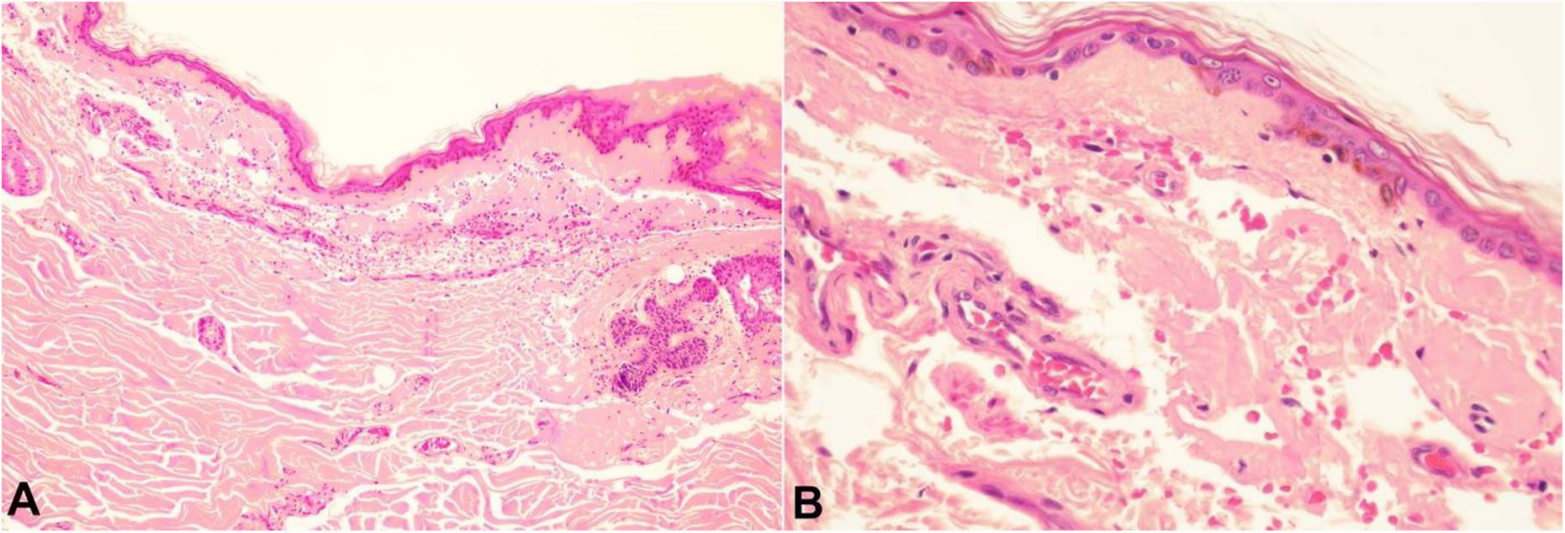
Photomicrographs of the skin. **A –** The epidermis is atrophic, and the dermis shows extensive amyloid deposits around the blood vessels, pilosebaceous units, and in the papillary dermis (H&E, 100x); **B –** High magnification view shows vascular damage and extravasation of red blood cells (H&E, 400x).

Microscopic sections of the skin showed massive amyloid deposition in the papillary dermis and around blood vessels, secondary atrophy of the epidermis, and extravasation of red blood cells, which causes the purpuric aspect of the clinical lesion and explains the friability of the skin (Figure 2).

**Figure 3:**
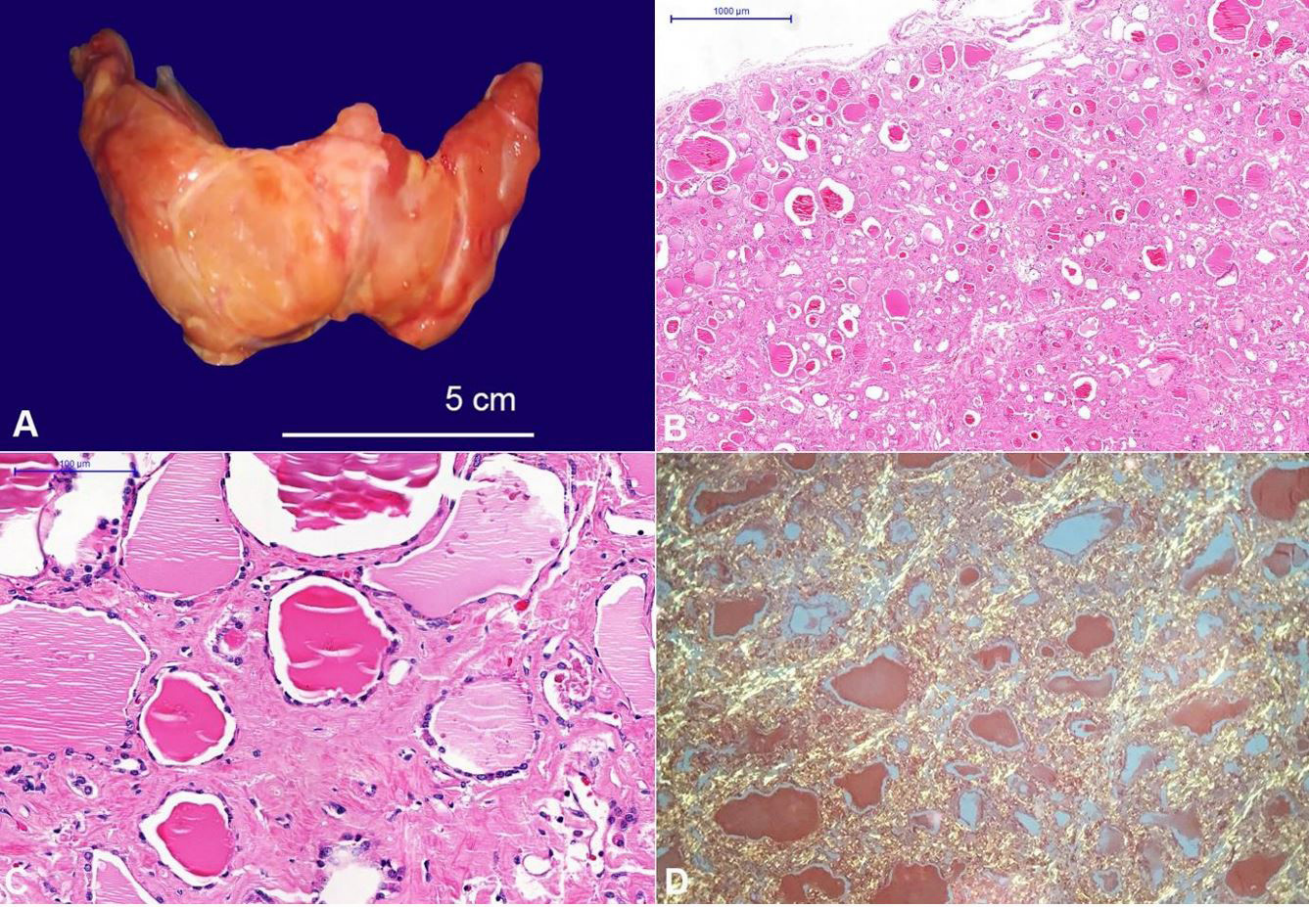
**A –** Gross examination of the thyroid: diffuse enlargement of the gland; **B, C**, and **D** – Photomicrographs of the thyroid: extensive amorphous hyaline material deposition in the interstitium causing marked follicular atrophy. The Congo red staining was strongly positive (B - H&E, 20x; C - H&E, 200x; and D - Congo red under polarized light, 100x).

The thyroid was diffusely enlarged and weighted 40 g (mean reference range [mRR]; 17 g) (Figure 3A). At the cut-surface, the parenchyma was firm and had a pale lardaceous appearance. No nodules were found. Microscopic sections of the gland showed diffuse interstitial amyloid deposition with secondary atrophy of the follicles (Figure 3B, C). After Congo red staining, the interstitial deposits had apple-green birefringence under polarized light (Figure 3D).

**Figure 4:**
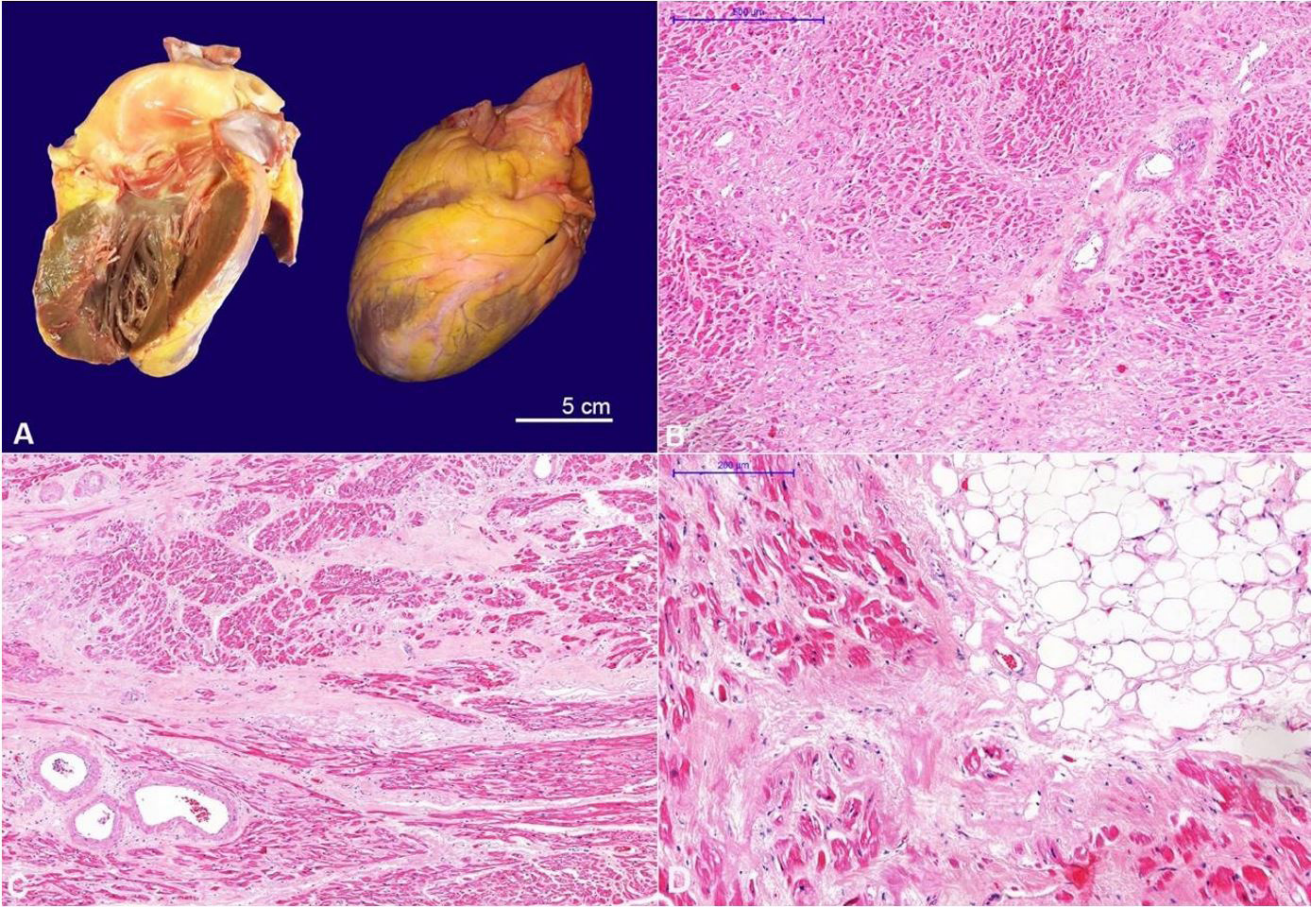
**A –** Gross examination of the heart: cardiomegaly with left ventricular hypertrophy; **B**, **C**, and **D –** Photomicrographs of the heart: amyloid deposits are seen in the interstitium between myocardial fibers, and in the epicardium around the adipocytes, known as “amyloid rings” pattern (B - H&E, 40x; C - H&E, 40x; and D - H&E, 100x).

At the opening of the thoracic cavity, a mild bilateral pleural effusion was drained. The heart weight was 450 g (mRR; 325 g), and concentric left ventricular hypertrophy was present (Figure 4A). The left ventricular wall measured 2.4 cm, the septum measured 2.0 cm (mRR, 1.15, and 1.35 cm, respectively), and subendocardial whitish areas were noted. Coronaries exhibited calcified atherosclerosis without critical stenosis, and the valves were normal. At the histological examination, amyloid was deposited in the blood vessels and intermingling the myocardiocytes accompanied by myocardial sclerosis (Figure 4B, C). The epicardial fat tissue exhibited amyloid deposition in a pattern of amyloid rings (Figure 4D).

At the opening of the trachea, a green-brownish fluid resembling enteric-stasis fluid was present. The lungs combined weight was 1638 g (mRR; 825 g). Their cut surface had bilateral basal congestion, and the airways were filled with dark brown secretion. The histological examination confirmed the presence of bilateral aspiration bronchopneumonia and diffuse alveolar damage with hyaline membranes.

**Figure 5:**
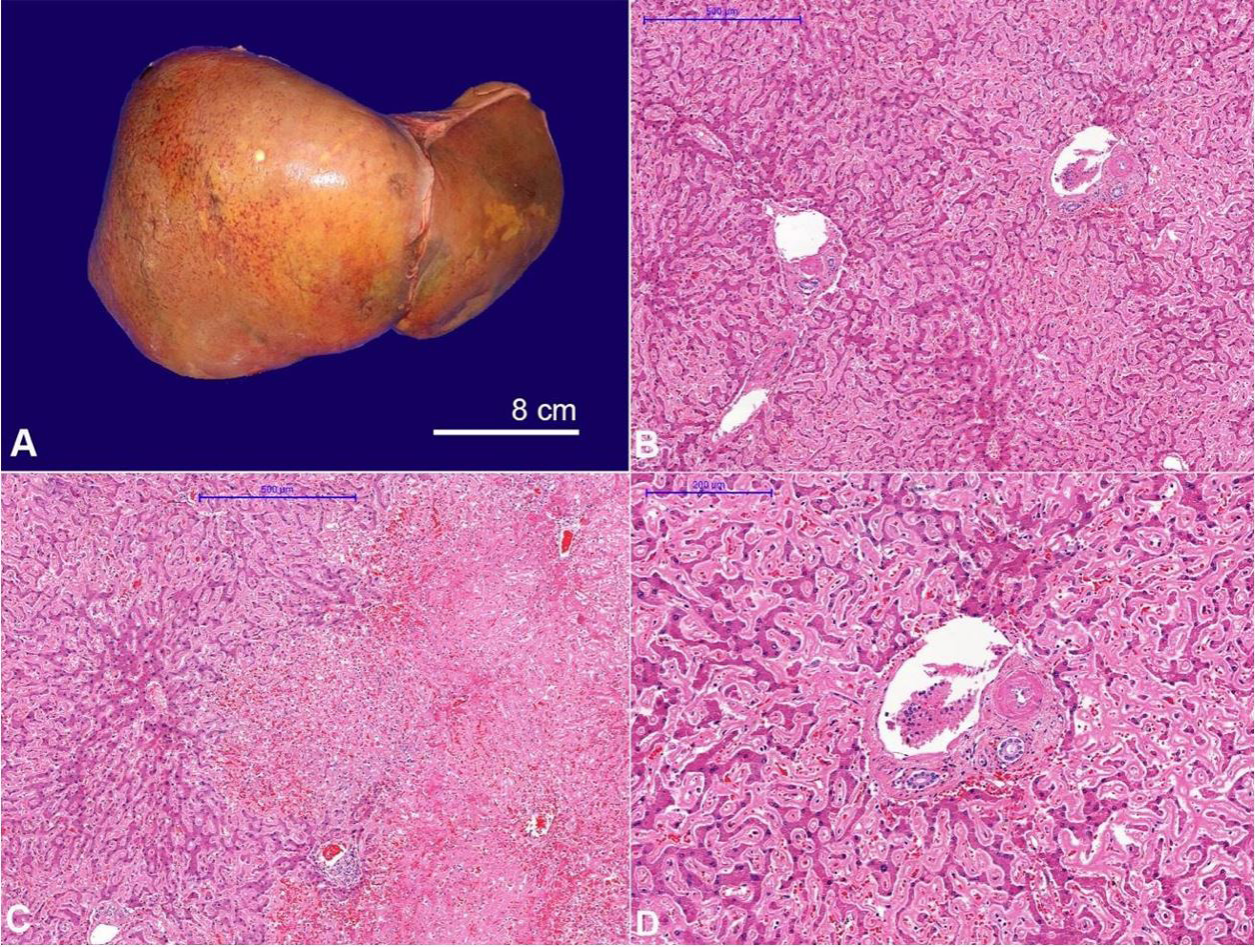
**A –** Gross view of the liver. Note the hepatic enlargement with hemorrhagic spots and yellowish necrotic foci; **B**, **C**, and **D** – Photomicrographs of the liver; **B** – shows diffuse portal tract, periportal tract, and sinusoidal amyloid deposition (H&E, 40x); **C –** viable hepatic parenchyma on the left and geographic hemorrhagic hepatic necrosis on the right side (H&E, 40x); **D –** High magnification of the portal tract showing the arteriolar wall thickening by amyloid deposits (H&E, 100x).

At the opening of the abdominal cavity, mild ascites was collected in the pouch of Douglas, while the peritoneal surface and the mesentery fat tissue were grossly normal. The liver was enlarged, weighed 1582 g (mRR; 1430 g), and the external surface was smooth and shiny with subcapsular hemorrhagic spots (Figure 5A). The consistency was soft and friable. The cut surface showed a pale subcapsular area in the right lobe and scattered yellowish focal lesions throughout the organ. On microscopy, amyloid deposits were conspicuously found in the sinusoids and the portal tracts, mainly in the wall of arterioles (Figure 5B, C, D).

**Figure 6:**
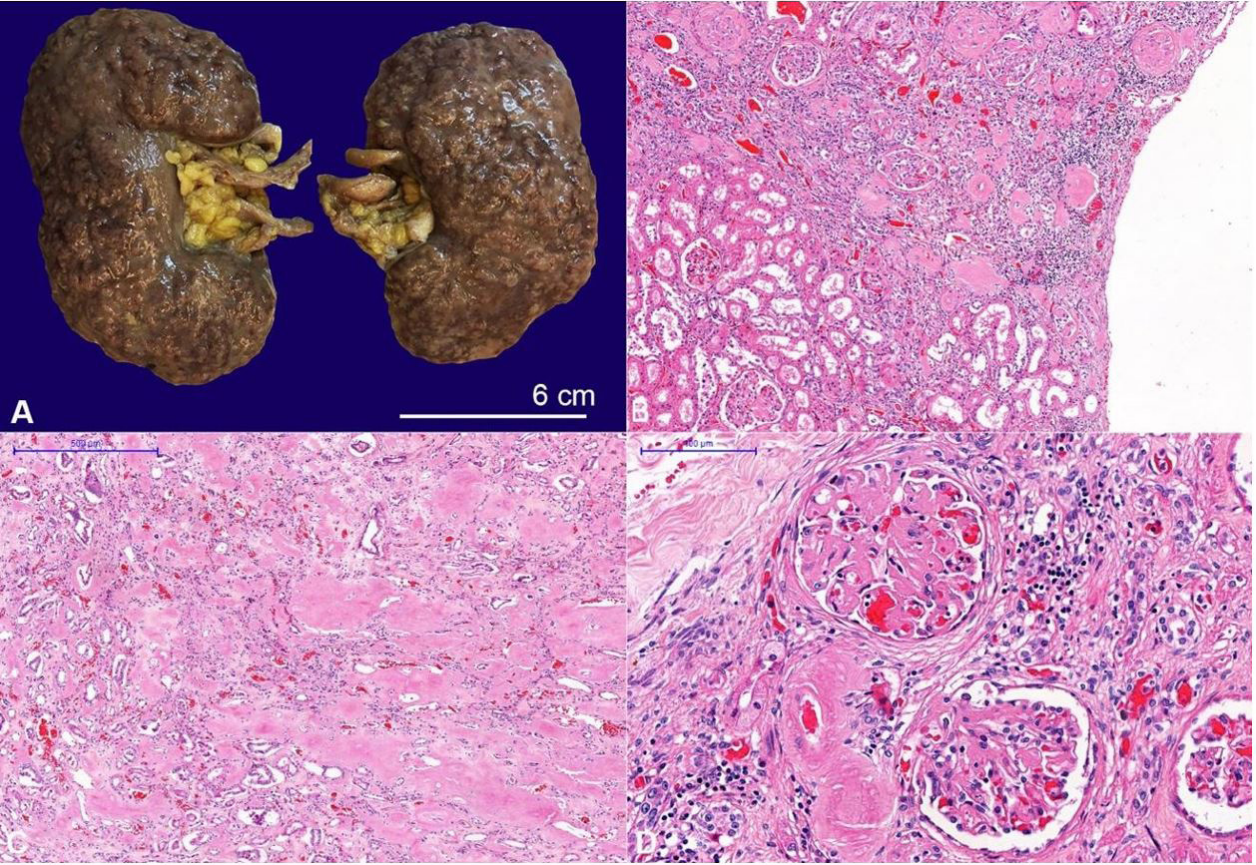
**A –** Gross view of the formalin-fixed kidneys, after the capsule detachment, showing coarse retractions throughout the cortical surface of both kidneys, causing volumetric retraction. **B**, **C**, and **D** – Photomicrographs of the kidney; **B –** Microscopic representation of the atrophic cortex due to amyloid deposition in the glomeruli and vessels (H&E, 50x); **C** and **D –** Conspicuous amyloid deposits in the tubular interstitium, glomerular mesangium and the arteriolar walls (C - H&E, 40X; and D - H&E, 400X).

The kidneys weighed 224g (mRR; 313g). On the external view, they appeared shrunken with a coarse and bilaterally granular external surface (Figure 6A). On histology, about 30% of the glomeruli were globally sclerosed, and the distribution of amyloid was heterogeneous. The cortical surface had wedge-shaped areas of diffuse glomerular and arteriolar amyloid deposition, interstitial fibrosis, atrophic tubules, and chronic inflammatory infiltrates, interchanged with areas of better-preserved parenchyma with fewer amyloid deposits, giving rise to the irregularity of the gross external view (Figure 6B). Amyloid deposits were also found in the interstitium of the renal medulla (Figure 6C) and almost all non-ischemic glomeruli (Figure 6D).

**Figure 7:**
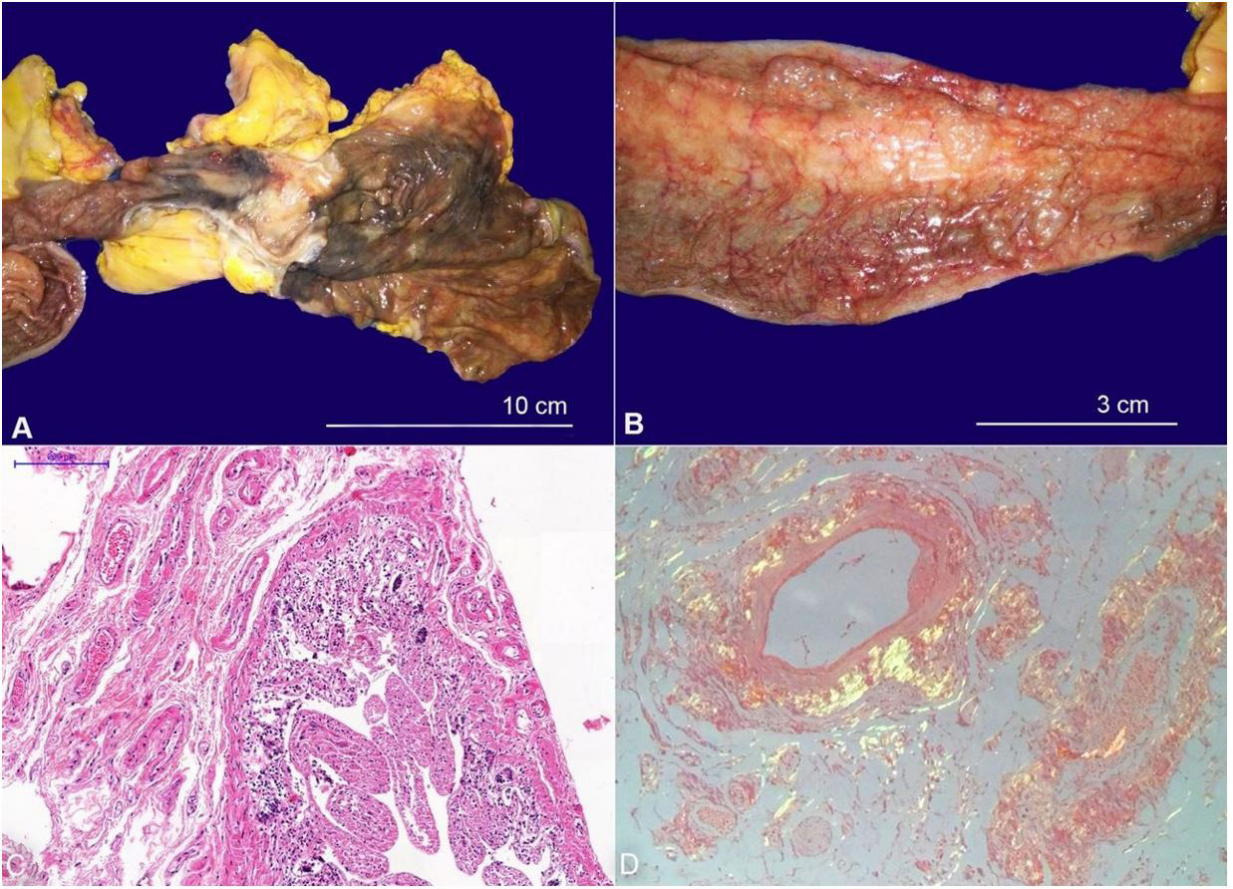
**A -** Gross view of the cecum with dark-green ischemic and necrotic mucosa; **B –** Gross view of a small bowel segment showing pneumatosis intestinalis; **C** and **D** – Photomicrographs of the small intestine wall and the mesentery. Note the coagulative necrosis of the enteric mucosa (C – H&E, 100x) and the amyloid deposits in the mesenteric blood vessels (D - Congo red under polarized light, 400x).

Regarding the alimentary tract, a diffuse dilatation of the stomach and bowels with scattered dark-green colored intestinal segments, emphysematous enteritis (pneumatosis intestinalis), and diffuse loss of the mucosal folds were observed (Figure 7A and 7B). Histologically, there was coagulative ischemic necrosis of the mucosa (Figure 7C). A large amount of amyloid protein deposits was found in the mesenteric and intestinal blood vessel walls (Figure 7D), which may have hampered the bowels’ perfusion and caused the ischemic findings.

**Figure 8:**
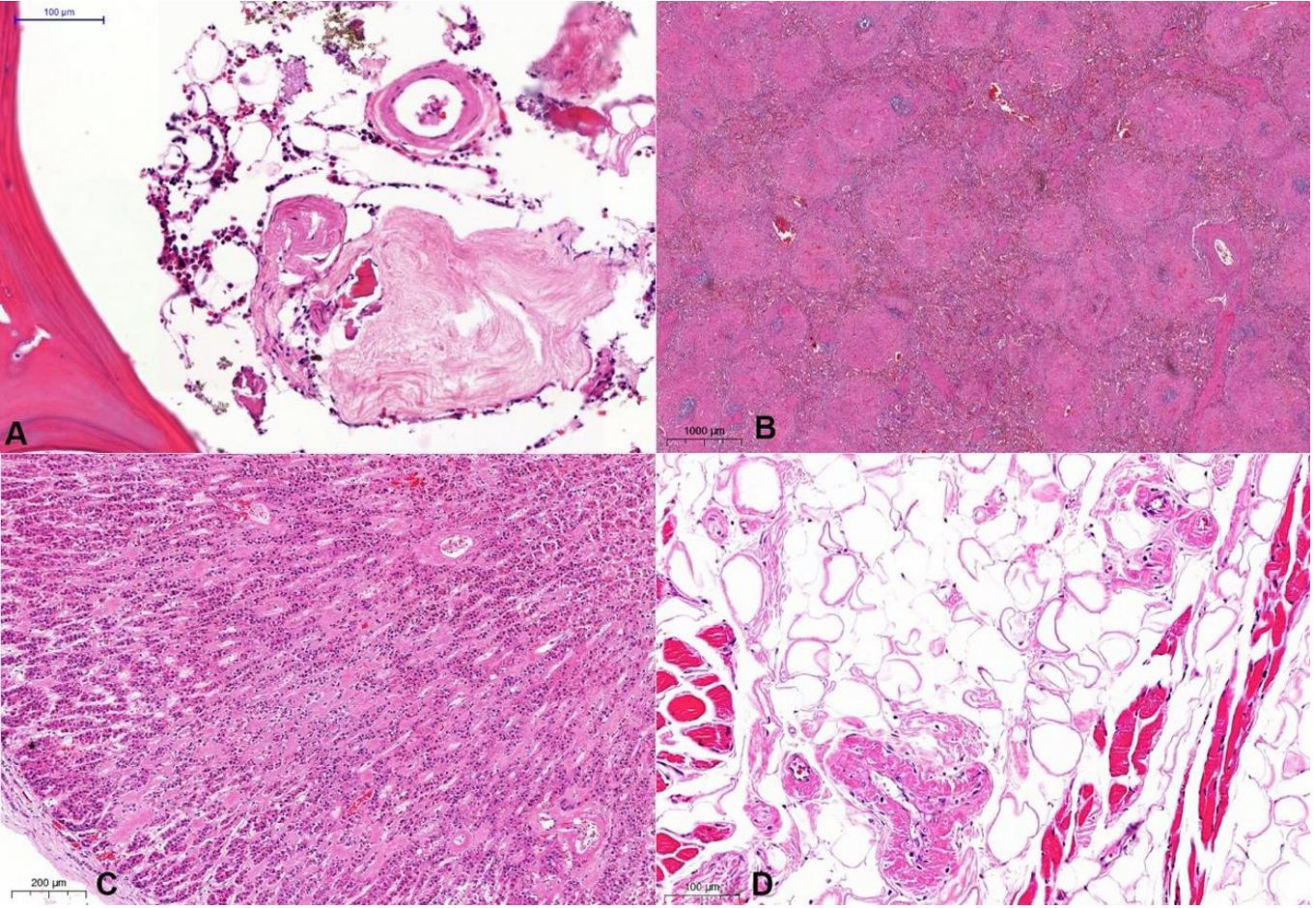
Photomicrographs of the bone marrow (A - H&E, 200x); the spleen (B - H&E, 10x); the adrenal (C - H&E, 50x) and the tongue adipose tissue (D - H&E, 100x). Note the hypocellular bone marrow with amyloid deposits in blood vessels and the interstitium; the micronodular aspect of the perifollicular amyloid deposition in the spleen; the interstitial deposits in the adrenal; and the diffuse “amyloid rings” pattern seen around the adipocytes in the tongue.

**Figure 9:**
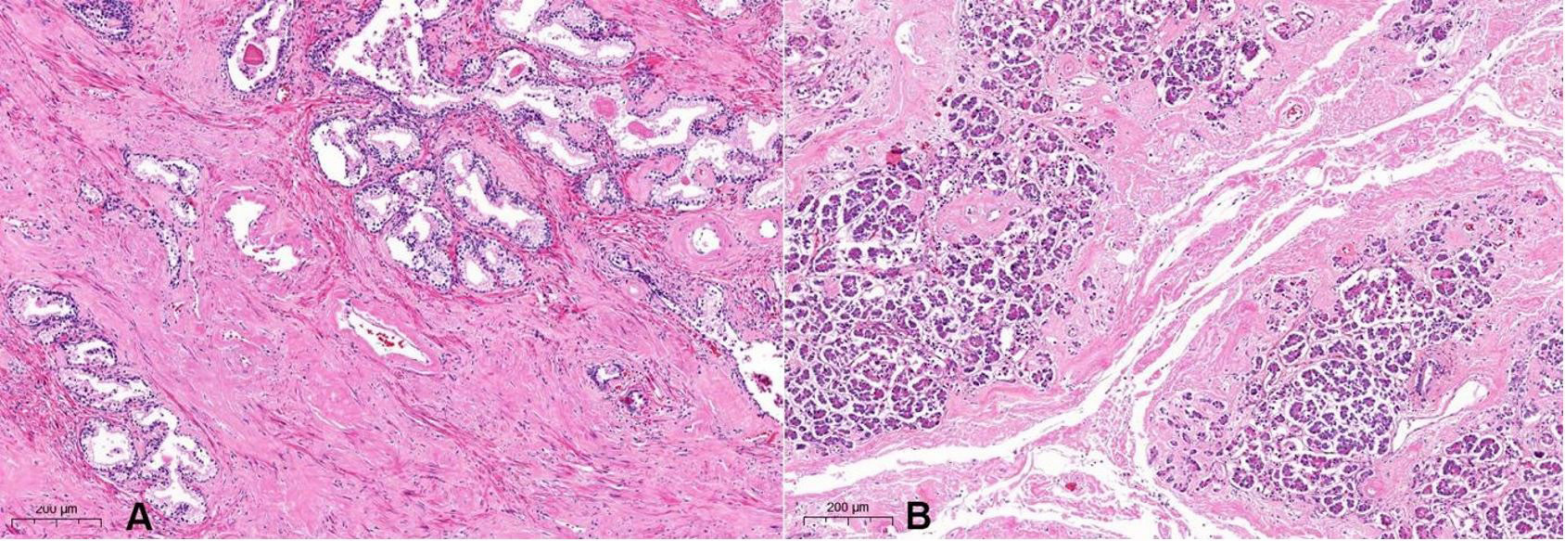
Photomicrographs of the prostate (A - H&E, 50x) and pancreas (B - H&E, 50x), showing amyloid deposits in the interstitium and blood vessels walls.

The histologic examination of the bone marrow showed hypocellularity and interstitial and perivascular amyloid deposits (Figure 8A), without morphological signs of myeloproliferative disorder. Immunohistochemical staining for CD3, CD20, CD138, Kappa, and Lambda were inconclusive. Massive amyloid deposition was also found in the enlarged spleen (weight= 198 g [RR 112 g]) (Figure 8B), the adrenals (Figure 8B), the tongue (Figure 8C), the prostate (Figure 9A), and the pancreas (Figure 9B). Gross and microscopic examinations of the central nervous system were unremarkable.

A sample of splenic tissue was subjected to 8-micron sections and Congo red staining. The positive regions of the tissue were dissected by laser microdissection microscopy and processed by peptide extraction. The obtained peptide extracts were then analyzed by liquid chromatography coupled to ultra-resolution tandem mass spectrometry. Mass spectrometry detected a peptide profile consistent with an immunoglobulin Kappa light chain, confirming the diagnosis of systemic AL (kappa light chain) amyloidosis.

## DISCUSSION

In our case, the definitive diagnosis of amyloid light-chain (AL) systemic amyloidosis was made through laser-capture microdissection and tandem mass spectrometry of a splenic tissue sample. Although the immunohistochemical panel performed on the bone marrow sample was inconclusive – likely due to post mortem artifacts – the diagnosis of primary amyloidosis was plausible since no clue of any other disease was found in the autopsy.

Amyloid deposits disrupt tissue structure leading to organ failure. Symptoms depend on the specific or major distribution of amyloid and, consequently, the organ impairment.^16^ In our case, diffuse amyloid deposition was found in the heart, liver, kidneys, spleen, prostate, thyroid gland, adrenals, and bone marrow. This extensive organic infiltration by the amyloid protein was the cause of the “in vivo” unexplained weight loss, purpura, liver failure, progressive renal insufficiency, and pancytopenia. Clinically, the patient also presented severe abdominal pain attributed to acute intestinal ischemia secondary to massive amyloid deposition in the intestinal blood vessels walls. Pneumatosis intestinalis has been previously described as a rare manifestation of gastrointestinal amyloidosis.^19^^-^^23^ Intestinal ischemia causes bowel dilation, aperistalsis, and bacterial translocation what explains the septic shock, unconsciousness or mental obnubilation, and consequent aspiration pneumonia. The sequence of these events explains the patient’s cause of death.

Weight loss is a frequent symptom of the systemic form of the disease, and it is likely multifactorial. Patients with systemic amyloidosis often present gastrointestinal involvement that may explain altered intestinal habits, lack of appetite, and latent hemorrhages due to direct mucous membrane impairment, aggression to the integrity of blood vessels, and changes of neuropathic nature.^24^ Secondary protein-losing enteropathy, associated with refractory nausea and vomiting, has been described in some patients and can lead to severe hypoalbuminemia.^25^ A complete clinical history of the patient was unavailable. However, as the emaciation was remarkable, we hypothesize that a chronic insufficient vascular supply to the gastrointestinal tract was the underlying cause of the patient's emaciation.

The involvement of the skin is more frequent in patients with primary systemic amyloidosis.^26^ Manifestations may vary from periorbital pinch purpura associated with minor trauma, a conspicuous sign named “racoon eyes”, to scattered or diffuse purpuric rash (petechiae and ecchymosis), as seen in our case.^27^ Two main factors which seem to play a role in the development of these lesions are: clotting alterations, namely a decrease in circulating coagulation factors and an increase in fibrinolysis; and subendothelial amyloid deposits in the blood vessels of the skin, favoring rupture and extravasation of red blood cells to the dermis.^28^

Thyroid goiter is one of the complications of systemic amyloidosis previously described in the literature and highlighted in this case. The gross examination shows the organ enlargement, and the cut surface is typically pale, firm, and has a waxy (or lardaceous) appearance. Histologically, the amyloid protein deposits are found in the interstitium, causing compression and secondary atrophy of the thyroid follicles.^29^^-^^32^ One of the lessons learned with this case is that the presence of thyroid goiter may favor the diagnosis of systemic amyloidosis over systemic vasculitis. Fast-growing goiter has been previously described as the first clinical manifestation of systemic amyloidosis in one patient.^33^

“Amyloid rings” is the amyloid deposits pattern in the adipose tissue (filling the intercellular space between the adipocytes). This finding was present, in our case, in the skin hypodermis, epicardium, tongue, and gastrointestinal tract subserosal fat. The pathologist should be aware of looking for this specific finding when examining a specimen from a periumbilical fat biopsy performed to investigate systemic amyloidosis. In clinical practice, the biopsy of subcutaneous fatty tissue (sensitivity around 75% and specificity around 90%) is usually preferred over trans-dermal core kidney biopsy (sensitivity close to 100%) due to being a lower risk procedure.^34^^-^^39^

## CONCLUSION

Despite the coexistence of a rich clinical feature and the availability of a diagnostic method, systemic amyloidosis remains misdiagnosed or undiagnosed. The unfamiliarity of the clinicians with this disease may explain the lack of early diagnosis. Widespread skin vascular lesions accompanied by polyvisceral involvement should always raise the hypothesis of systemic amyloidosis. Other manifestations of the disease include thyroid goiter, hepatosplenomegaly, and even pneumatosis intestinalis. In this case, a post-mortem examination was essential to clarify the cause of death, and therefore a precise death certificate. Additionally, the autopsy offered information better to understand the patient’s symptoms and laboratory findings. THINK ABOUT AMYLOIDOSIS!
